# Coarse-grained statistics for attributing criticality to heterogeneous neural networks

**DOI:** 10.1186/1471-2202-12-S1-P235

**Published:** 2011-07-18

**Authors:** Thomas Gregory Corcoran, Andy Philippides, Thomas Nowotny

**Affiliations:** 1 University of Sussex, Brighton, UK

## 

Multi-scale modelling has great use in computational neuroscience [1]. This typically involves a stage of coarse graining. However, the assumptions (e.g. that the sum of ensemble activities are dominated by the mean and fluctuations are Gaussian) that underlie a coarse-grained description have yet to be qualified, especially in the biological context where intrinsic diversity can contribute to complex activity patterns [2]. Recent work has reported on the effects that intrinsic properties can have on overall network activity [3], while parametric variance is proposed to have self-regulatory and tuning effects [4,5].

Here, we report on multi-scale analysis of data from large-scale neuronal network simulations which involves a coarse-graining procedure of the observed spike patterns. At each scale of coarse-graining, observables are calculated and the scale-dependence of summary statistics is determined. We observe whether the activity forms contiguous events, also known as avalanches, and measure their size. This study differs from others in that it uses coarse graining in both time and space.

Our model system contains a heterogeneous population of adaptive exponential integrate-and-fire model neurons with various heterogeneities on a spatial sheet with toroidal boundary conditions. We then examine locally averaged spike rates calculated across space and time on different scales to form a hierarchical description. We then analyse these coarse grained variables in a detrended fluctuation analysis (DFA).

We find that there is an appropriate scale for intermittency in the coarse-grained observables. We characterise the properties of contiguous events in this regime and qualify the hypothesis of a critical phase transition.

In a second analysis, we study spatial correlations in the coarse grained activity and their scaling properties confirming our findings using DFA.

Our numerical "renormalisation group" analysis allows us to determine those properties which are most relevant to meso/macroscopic statistics of spiking neural networks, while at the same time providing a means to formally investigate brain criticality.
